# Association between triglyceride glucose index and arterial stiffness and coronary artery calcification: a systematic review and exposure-effect meta-analysis

**DOI:** 10.1186/s12933-023-01819-2

**Published:** 2023-05-13

**Authors:** Fuwei Liu, Qin Ling, Shaofeng Xie, Yi Xu, Menglu Liu, Qingwen Hu, Jianyong Ma, Zhiwei Yan, Yan Gao, Yujie Zhao, Wengen Zhu, Peng Yu, Jun Luo, Xiao Liu

**Affiliations:** 1 Present Address: Department of Cardiology, The Affiliated Ganzhou Hospital of Nanchang University, Jiangxi, China; 2 The Second Clinical Medical College of Nanchang University, The Second Affiliated Hospital of Nanchang University, Nanchang, 330006 China; 3 Department of Cardiology, Seventh People’s Hospital of Zhengzhou, Zhengzhou, Henan China; 4 Department of Pharmacology and Systems Physiology, University of Cincinnati College of Medicine, Cincinnati, USA; 5 Department of Sports Rehabilitation, College of Human Kinesiology, Shenyang Sport University, Shenyang, China; 6 Department of Cardiology, The First Affiliated Hospital of Sun Yat-Sen University, Guangzhou, Guangdong China; 7 Department of Endocrine, The Second Affiliated Hospital of Nanchang University, Nanchang, Jiangxi China; 8 Department of Cardiology, The Sun Yat-Sen Memorial Hospital of Sun Yat-Sen University, Guangzhou, Guangdong China; 9 Provincial University Key Laboratory of Sport and Health Science, School of Physical Education and Sport Sciences, Fujian Normal University, Fuzhou, Fujian China

**Keywords:** Triglyceride and glucose index, Arterial stiffness, Coronary artery calcification, Exposure-effect, Meta-analysis

## Abstract

**Background:**

The triglyceride and glucose (TyG) index has been linked to various cardiovascular diseases. However, it's still unclear whether the TyG index is associated with arterial stiffness and coronary artery calcification (CAC).

**Methods:**

We conducted a systematic review and meta-analysis of relevant studies until September 2022 in the PubMed, Cochrane Library, and Embase databases. We used a random-effects model to calculate the pooled effect estimate and the robust error meta-regression method to summarize the exposure-effect relationship.

**Results:**

Twenty-six observational studies involving 87,307 participants were included. In the category analysis, the TyG index was associated with the risk of arterial stiffness (odds ratio [OR]: 1.83; 95% CI 1.55–2.17, I^2^ = 68%) and CAC (OR: 1.66; 95% CI 1.51–1.82, I^2^ = 0). The per 1-unit increment in the TyG index was also associated with an increased risk of arterial stiffness (OR: 1.51, 95% CI 1.35–1.69, I^2^ = 82%) and CAC (OR: 1.73, 95% CI 1.36–2.20, I^2^ = 51%). Moreover, a higher TyG index was shown to be a risk factor for the progression of CAC (OR = 1.66, 95% CI 1.21–2.27, I^2^ = 0, in category analysis, OR = 1.47, 95% CI 1.29–1.68, I^2^ = 41% in continuity analysis). There was a positive nonlinear association between the TyG index and the risk of arterial stiffness (P_nonlinearity_ < 0.001).

**Conclusion:**

An elevated TyG index is associated with an increased risk of arterial stiffness and CAC. Prospective studies are needed to assess causality.

**Graphical Abstract:**

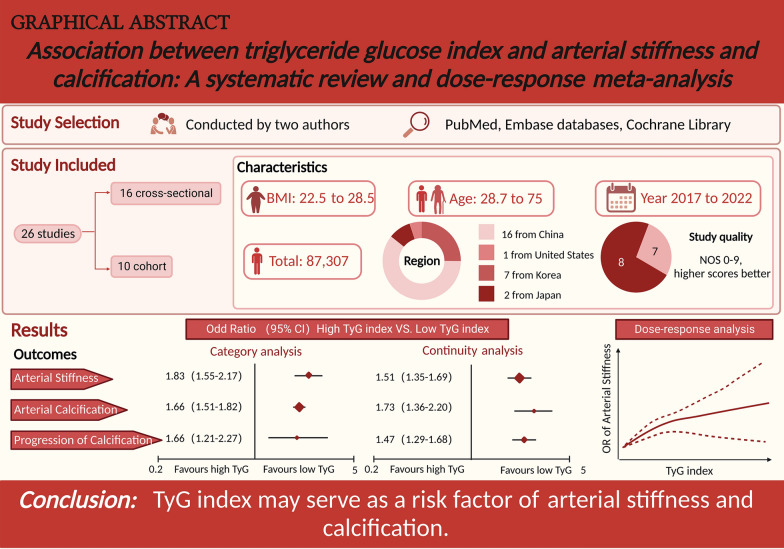

**Supplementary Information:**

The online version contains supplementary material available at 10.1186/s12933-023-01819-2.

## Introduction

Cardiovascular disease (CVD) accounts for nearly a third of all deaths worldwide annually [1], with complex structural and functional changes occur in the arterial system, characterized by coronary artery calcification (CAC) and gradually increasing stiffness of vessels [2]. Recognized as a marker of adverse cardiovascular outcomes, CAC is concomitant with the development of advanced atherosclerosis [3]. Arterial stiffness can cause changes in both the extracellular matrix of elastic arteries and the mechanical properties of the vascular wall, thereby activating the atherosclerotic process [4]. Both are independent predictors for cardiovascular mortality, whose processes reinforce one another, creating vicious cycles in the body [5]. Calculated by triglyceride and glucose, the triglyceride glucose (TyG) index was newly proposed as a reliable and applicable tool for predicting insulin resistance (IR) [6] and may be more reliable than the commonly used indicator for clinical assessment, homeostasis model assessment-insulin resistance (HOMA-IR), in terms of both sensitivity and specificity [7].

Studies have shown that a higher TyG index is associated with an increased risk of cardiovascular events and mortality in the general population [8–10]. It is also associated with subclinical cardiovascular diseases, such as arterial stiffness and CAC [11]. Arterial stiffness and CAC are well-known risk factors for cardiovascular events, such as myocardial infarction [12] or stroke [13]. For example, Wu et al. [14] concluded that participants with a higher TyG index should be aware of the subsequent risk of arterial stiffness progression, and an independent association between the TyG index and extensive abdominal aortic CAC was found by Chen et al. [15]. These findings suggest that the TyG index may serve as a marker independent of traditional risk factors for arterial stiffness and CAC. However, there is no systematic review that critically evaluated their association. Therefore, we aimed to conduct a meta-analysis of observational studies to evaluate the association between the TyG index and arterial stiffness and CAC.

## Methods:

### Protocol and registration

The protocol was registered with PROSPERO (International prospective register of systematic reviews). https://www.crd.york.ac.uk/PROSPERO/-registration number- CRD42022360981). We reported this systematic review and meta-analysis based on the guidelines of the Preferred Reporting Items for Systematic Reviews and Meta-analysis (PRISMA) [16], which can be seen in Additional file 1: Table S1.

### Literature search

Studies were identified through a systematic search of the electronic databases of Embase (https://www.embase.com/), Cochrane Library (http://www.cochranelibrary.com), and PubMed (https://pubmed.ncbi.nlm.nih.gov/) until September 9, 2022, for the most exhaustive literature review. The search terms used were (‘TyG index’, ‘triglyceride-glucose index’, ‘triglyceride and glucose index’ or ‘triglyceride glucose index’) and (‘vascular stiffness’ or ‘brachial-ankle pulse wave velocity’ or ‘CAC’ or ‘coronary artery calcium score’) to identify all eligible reports. Additional file 1: Table S2 describes the full search terms used in each database searched.

### Study selection

Two investigators (Q-L and X-Y) independently finished the whole process from the literature search and selection to data analysis. We used Endnote X9 software (Tomson Reuters, New York, NY, USA) to organize all studies. After removing the duplicates automatically and manually, we performed a preliminary screening of the relevant literature by examining the titles and abstracts. When the article or additional information was unavailable, the corresponding author was contacted to obtain information. Afterwards, we performed a full-text reading of the initially screened literature to identify the final available studies. Any discrepancies in this process were resolved by the third reviewer (P-Y).

The inclusion criteria for the studies were as follows according to the PICOS: (1) types of participants: adult (age > 18 years); (2) exposure and comparator: high versus low TyG index level; (3) outcomes: evaluated the association between TyG index and risk or progession of arterial stiffness or CAC; (4) types of studies: observational studies published as full-length articles; and (5) reported the estimated effect for this association with multivariate analysis. We excluded studies if they were reviews, meta-analyses, abstract-only articles, or focused on other outcomes. We also excluded studies with data that could not be extracted or were not reported. We considered a certain degree of elevated CACS or microcalcifications grows into larger calcium fragments [3] as “progression of CAC.

### Data extraction and quality assessment

Two review authors (Q-L and X-Y) independently extracted the relevant information from the eligible studies, and any discrepancies were resolved by consensus. We used predesigned Microsoft Excel 2019 (Microsoft Corporation, Redmond, WA, USA) for data extraction, and the following data were extracted from the studies: (1) first author name; (2) publication year; (3) country or region; (4) study type; (5) duration of follow-up; (6) basic characteristics (sample size, age, body mass index-BMI and so on); (7) source of participants; (8) outcome; (9) adjustments; (10) hazard ratio (HR) or relative risk (RR) or odds ratio (OR) with 95% confidence interval (CI) from the most adjusted model.

For the included cohort studies, the quality and strength of the evidence for each outcome were assessed by the Newcastle Ottawa Quality Assessment Scale (NOS). This is based on the selection and comparability of studies and the determination of the exposure or outcome [17]. Studies gaining more than 6 stars out of a possible 9 were considered to have a low risk of bias. Additionally, we used the Joanna Briggs Institute's critical appraisal checklist to analyse the quality of the included cross-sectional studies [18].

### Statistical analysis

The TyG index was calculated as Ln(Fasting Triglycerides [mg/dL] × Fasting Plasma Glucose [mg/dL]/2) [19]. Since the meta-analysis included cohort and cross-sectional studies, HRs were treated as ORs. For articles that reported the TyG index as a categorical variable, we extracted the effect estimates of the highest TyG index group versus the lowest TyG index group. In the analysis of continuous variables, the effect estimates of the TyG index per 1-unit increment were evaluated. In cases where continuous data were not available, we used variance-weighted least-squares regression analysis to compute the linear trend [20]. We pooled the data with RevMan software, version 5.3 (The Cochrane Collaboration 2014, Nordic Cochrane Center Copenhagen, Denmark) and Stata software (Version 16.0, Stata Corp LP, College Station, TX, USA), using the generic inverse variance method of Der Simonian and Laird in a random-effects model [20]. We calculated the proportion of the total variability in the effect estimates due to heterogeneity (I^2^), and also estimated the variance between the studies (τ^2^). For exposure-effect analysis between the TyG index and arterial stiffness, we used the robust error meta-regression method [21], which required at least two categories with corresponding effect estimates.

We assessed the heterogeneity across the included articles using Cochrane's Q test (τ^2^). I^2^ was used to assess inconsistency among our findings. We performed sensitivity analysis using Stata 16.0 (StataCorp, US) to test the stability of the results. We also analyzed the publication bias for the results with more than 10 eligible articles by funnel plot, Egger’s test, and Begg’s test. A statistically significant effect was considered when the P value was less than 0.05.

## Results

### Literature search

**Fig. 1:**
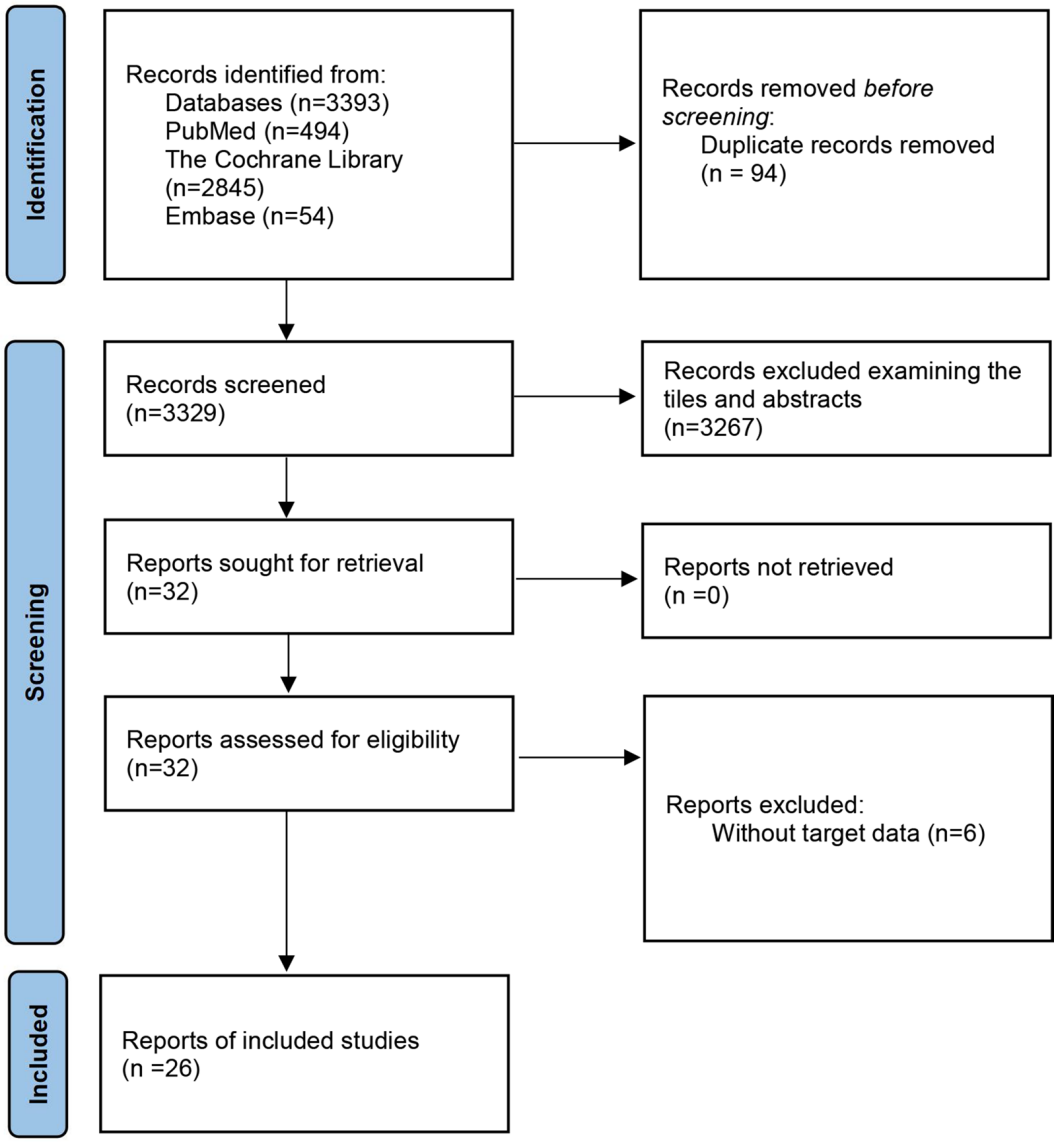
Flow chart of the study selection process

We conducted a comprehensive literature search following our search strategy, which included searching through PubMed (494 articles), The Cochrane Library (2845 articles), and Embase (54 reports), totaling 3393 reports. After removing duplicates (94 reports), we screened the remaining 32 articles by reading the title and abstract, and assessed six additional articles for lacking target data (n = 6) (Fig. 1) [11, 22–26]. Finally, ten cohort studies[14, 27–35] and sixteen cross-sectional [9, 15, 36–49] studies were included in the present research, while the detailed reasons for the excluded articles are listed in Additional file 1: Table S3.

### Study characteristics and quality evaluation

Table 1 presents the baseline characteristics of the included studies, which were published between 2017 and 2022, with a sample size ranging from 180 to 13,706 and a total of 87,307 participants. Among the included studies, 15 were from China[9, 14, 15, 28, 29, 31, 33, 34, 36, 40, 42, 44, 45, 47, 50], seven were conducted in Korea [27, 30, 34, 35, 37–39], three were from [42, 48, 49] Japan [41, 47, 49], and only one originated in non-Asia (United States) [43]. The mean age of participants ranged from 28.7 to 75 years, and the BMI ranged from 22.5 to 28.5 kg/m^2^. Although the included studies varied in their endpoint measurement methods, arterial stiffness was mostly diagnosed using brachial-ankle pulse wave velocity (baPWV), and arterial calcification was mostly graded using coronary artery calcium score (CACS).

**Table 1:** Characteristics of included studies in this meta-analysis

References (first author, year, country/region)	Source of participants	Participant characteristics	Study design	N	Mean age (years), Male (%)	Mean BMI (kg/m^2^)	Categories of TyG	Outcome/measurement	OR/HR (95% CIs)	Follow-Up Period	Adjustments
Arterial stiffness											
Lee, 2018, Korea	Gangnam Severance Hospital Health Promotion Center	General population	Cross-sectional study	3587	52.0, 57.5	23.4	Men: 7.73 8.20 8.57 9.16 Women: 7.73 8.20 8.57 9.16	Arterial stiffness/baPWV	Ref. 1.55 (1.02–2.36) 1.81 (1.20–2.71) 2.92 (1.92–4.44) Ref. 1.28 (0.80–2.03) 1.56 (0.97–2.52) 1.84 (1.15–2.96)	NR	Age, SBP, BMI, LDL-C, HDL-C, diabetes, and menopause (women)
Zhao, 2019, China	The Northern Shanghai Study	General population	Cross-sectional study	2830	71.5, 44.5	24.0	7.7 8.52 8.89 10.36	Arterial stiffness/baPWV	Ref. 1.30 (1.03–1.65) 1.48 (1.15–1.91) 1.39 (1.05–1.84)	NR	Age, sex, BMI, WC, smoking, hypertension, family history of premature CVD, diabetes, LDL-C, HDL-C, insulin therapy and statin therapy
Poon, 2020, USA	The Atherosclerosis Risk in Communities Study	General population	Cross-sectional study	2571	75.0, 37.0	27.0	Continuous variable	Arterial stiffness/NR	1.21 (1.11–1.32)	24 years	Age, sex (except for gender-specific estimates), and race or study site
Nakagomi, 2020, Japan	Chiba Foundation for Health Promotion & Disease Prevention	General population	Cross-sectional study	2818	38.9, 61.0	22.5	Continuous variable	Arterial stiffness/baPWV	1.53 (1.16–2.02)		Age, SBP, BMI, HbA1c, FBG, LDL-C, HDL-C, UA, smoking, and alcohol intake
Su, 2021, China	Wanshou Road Community of Haidian District in Beijing	Chinese older adults	Cross-sectional study	2035	71.3, 60.4	25.0	8.22 8.65 8.99 9.58 Continuous variable	Arterial stiffness/baPWV	Ref. 1.18 (0.82–1.68) 1.27 (0.87–1.87) 1.78 (1.12–2.81) 1.32 (1.09–1.60)	NR	Age, sex, BMI, WC, SBP, DBP, TC, LDL-C, HDL-C, UA, eGFR, smoking, alcohol intake, CHD, hypertension, diabetes, antiplatelet drugs, AHD, hypoglycemic therapy, and lipid-lowering therapy
Wang, 2021, China	National Metabolic ManAgement Center (MMC) in Ruijin Hospital, Shanghai Jiao Tong University School of Medicine	Patients with T2D	Cross-sectional study	3185	54.6, 61.4	25.8	8.45 9.09 9.94 Continuous variable	Arterial stiffness/baPWV	Ref. 1.40 (1.06–1.83) 1.49 (1.09–2.04) 1.40 (1.16–1.70)		Age, sex, SBP, BMI, WC, HbA1c, diabetes, LDL-C, HDL-C,WBC counts, smoking, alcohol intake, lipid lowering Agents, AHD, insulin therapy, non-insulin hypoglycemic Agents
Pan, 2021, China	Kunshan Hospital Affiliated to Jiangsu University	Patients with T2D	Cross-sectional study	4721	59.6, 53.6	25.3	Continuous variable	Arterial stiffness/baPWV	1.38 (1.21–1.57)	NR	Age, sex, BMI, HbA1c, and smoking
Zhang, C, 2022, Japan	TheDRYAD database	General population	Cross-sectional study	912	51.1, 64.9	NR	Tertile 1 Tertile 2 Tertile 3 Continuous variable	Arterial stiffness/baPWV	Ref. 1.60 (0.96–2.75) 1.78 (0.93–3.39) 1.65 (1.08–2.54)	NR	Age, BMI, SBP, HDL-C, TC, eGFR, UA, fatty liver, smoking, alcohol intake, and physical activity
Yang, 2022, Japan	DATADRYAD database	General population	Cross-sectional study	912	51.1, 64.9	23.1	7.66 8.27 8.88 Continuous variable	Arterial stiffness/baPWV	Ref. 1.43 (0.93–2.19) 1.78 (1.08–2.95) 1.57(1.14–2.18)	NR	Age, sex, BMI, SBP, DBP, HDL-C, eGFR, and fatty liver
Zhang, 2022, China	Pidu District People’s Hospital,	Non-hypertensive Chinese	Cross-sectional study	3265	40.2, 47.0	22.5	7.81 8.16 8.55 8.98	Arterial stiffness/CAVI	Ref. 1.47 (0.87–2.46) 1.82 (1.10–3.01) 2.35 (1.41–3.90)	4.71 years	Age, sex, SBP, DBP, BMI, smoking, alcohol intake, diabetes, prehypertension, hyperuricemia, and renal dysfunction
Li, 2020, China	China H-type Hypertension Registry Study in Wuyuan	General population	Cross-sectional study	4718	64.4, 49.7	23.2	7.77 8.59 9 10.44 Continuous variable	Arterial stiffness/baPWV	Ref. 0.59 (0.33–0.85) 0.89 (0.61–1.18) 1.56 (1.25–1.88) 1.02 (0.83–1.20)	NR	Age, sex, SBP, DBP, BMI, WC, smoking, alcohol intake, physical activity, education, SUA, serum homocysteine, LDL-C, HDL-C, eGFR, diabetes, AHD, antiplatelet drugs
Wu, 2021, China	Kailuan cohort (Kailuan General Hospital and 10 affiliated hospitals)	General population	Prospective cohort study	5348	46.9, 59.5	24.6	7.44 8.27 8.73 11.08 Continuous variable	Arterial stiffness/baPWV	Ref. 1.27 (1.00–1.62) 1.69 (1.35–2.12) 1.58 (1.25–2.01) 1.22 (1.10–1.35)	5 years	Age, sex, BMI, hs-CRP, MAP, smoking, alcohol intake, physical activity, and diabetes
Yan, 2022, China	Hanzhong Adolescent Hypertension Cohort study	General population	Cross-sectional study	180	28.7, 60.0	22.6	Low-stable Moderate High-increasign	Arterial stiffness/baPWV	Ref. 2.51 (0.85–7.39) 2.76 (1.40–7.54)	12 years	Age, sex, physical activity, and hypertension
Han, 2022, China	Beijing Health Management Cohort (BHMC) study	General population	Prospective cohort study	3048	56.0, 75.8	25.5	8.2 8.57 8.96 9.36	Arterial stiffness/baPWV	Ref. 0.87 (0.69–1.12) 0.96 (0.73–1.26) 1.23 (0.91–1.64)	8 years	Age, sex, BMI, MAP, LDL-C, HDL-C, education, smoking, alcohol intake, physical activity, sleep duration, excessive salt intake, anemia, and medication history
Guo, 2021, China	Health Promotion Center of the First Affiliated Hospital of Nanjing Medical University	General population	Retrospective cohort study	13,706	49.4, 55.7	24.6	Continuous variable	Arterial stiffness/baPWV	1.51 (1.37–1.67)	NR	Age, BMI, smoking, pulse pressure, HbA1c, TC, LDL-C, HDL-C, UA and AHD
Ji, 2022, China	Gucheng and Pingguoyuan communities of Shijingshan District in Beijing	General population	Cross-sectional study	6015	62.4, 34.3	25.2	8.28 8.77 9.26 Continuous variable	Arterial stiffness/baPWV	Ref. 1.32 (1.11, 1.58) 1.79 (1.48, 2.17) 1.61(1.42–1.84)	7 years	Age, sex, SBP, DBP, BMI, smoking, alcohol intake, LDL-C, HDL-C, eGFR, CHD, stroke, AHD, HGD, and LLD
CAC											
Kim, 2017, Korea	the Gangnam Severance Hospital Health Promotion Center	General population	Cross-sectional study	4319	53.4, 53.8	23.6	7.75 8.22 8.59 9.157 Continuous variable	CAC/CACS	Ref. 1.18 (0.75–1.85) 1.28 (0.85–1.99) 1.95 (1.23–3.11) 1.59 (1.16–2.18)	NR	Age, sex, SBP, BMI, LDL-C, HDL-C, smoking, alcohol intake, and physical activity
Kim, J, 2017, Korea	the Kangbuk Samsung Health Study	General population	Cross-sectional study	4420	41.2, 80.4	24.3	1st tertile 2nd tertile 3rd tertile	CAC/CACS	Ref. 1.14 (1.04–1.26) 1.49 (1.35–1.64)	NR	Age, sex, SBP, LDL-C, smoking, and physical activity
Won, 2018, Korea	NR	General population	Retrospective cohort study	2840	57.5, 51.1	24.5	Continuous variable	CAC/CACS	1.45 (1.03–2.04)	NR	NR
Chen, 2021, China	NHANES 2013–2014 cohort	General population	Cross-sectional study	1419	57.5, 48.3	28.5	8.22 8.56 9.1 Continuous variable	ACC/X-ray absorptiometry	Ref. 1.44 (0.91–2.28) 1.80 (1.11–2.94) 1.41 (1.04–1.91)	NR	Age, sex, BMI, hypertension, diabetes, high cholesterol, smoking, metabolic equivalent, UA, total 25-hydroxyvitamin D, calcium, race, phosphorus, eGFR and NLR
Si, 2021, China	NR	General population	Retrospective cohort study	697	60.0, 47.8	25.0	Continuous variable	CAC/CACS	2.12 (1.24–3.65)	NR	Age, hypertension, T2D, and MLR
Wang,2022, China	Second Hospital of Shandong University	Patients with acute coronary syndrome	Retrospective cohort study	935	65.0, 70.0	27.7	Continuous variable	CAC/CACS	2.90 (2.53–4.60)	34.5 months	Age, sex, SBP, DBP, BMI, AHD, HGD, LLD, body weight, WC, PPD, heart rate, FBG, HbA1c, LVEF, diabetes, hypertension, hyperlipidemia, smoking, alcohol intake, WBC, TC, LDL-C, Cr, UA, Cys C, homocysteine, D-dimer, troponin I, and BNP
Song, 2022, China	Coronary Artery Calcification (KOICA) registry	General population	Retrospective cohort study	5775	49, 82.6	24.4	Continuous variable	CAC progression/CACS	1.57 (1.36–1.81)	3.5 years	Age, sex, BMI, SBP, DBP, HDL- C, LDL-C, smoking, 10-year ASCVD risk, and serum creatinine
Park, 2019, Korea	the Gangnam Severance Hospital Health Promotion Center in Seoul	General population	Retrospective cohort study	1175	51.8, 71.1	24.2	7.94 8.54 9.18	CAC progression/CACS	Ref. 1.15(0.78–1.71) 1.82(1.20–2.77)	4.2 years	SBP, BMI, LDL-C, HDL-C, smoking, alcohol intake, physical activity, diabetes, hypertension, use of statins and aspirin, and baseline ln(CACS + 1)
Won, 2020, Korea	Korea Initiatives on Coronary Artery Calcification	General population	Retrospective cohort study	4731	51.7, 84.2	24.6	Continuous variable	CAC progression/CACS	1.37 (1.18–1.59)	3.5 years	Age, sex, BMI, hypertension, diabetes, hypercholesterolemia, smoking, and serum creatinine level
Cho, 2020, Korea	Asan Medical Center (AMC; Seoul, Korea)	General population	Prospective cohort study	1145	54.2, 81.7	25.0	Quartile 1 Quartile 2 Quartile 3 Quartile 4	CAC progression/CACS	Ref. 1.65 (1.06–2.57) 1.26 (0.78–2.02) 1.46 (0.90–2.38)	3 years	Age, sex, SBP, LDL-C, HDL-C, smoking, alcohol intake, physical activity, CACS, and follow-up interval

*BMI* body mass index, *TyG* triglyceride and glucose index, *OR* odds ratio, *HR* hazards ratio, *CI* confidence interval, *NR* not reported, *CAC* coronary artery calcification, *ACC* abdominal aortic calcification, *baPWV* brachial-ankle pulse wave velocity, *CAVI* cardio–ankle vascular index, *CACS* coronary artery calcium score, *T2D* type 2 diabetes, *SBP* systolic blood pressure, *LDL-C* low density lipoprotein cholesterol, *HDL-C* high density lipoprotein cholesterol, *WC *waist circumference, *CVD* cardiovascular disease, *CACS* coronary artery calcium score, *DBP* diastolic blood pressure, *SUA* serum uric acid, *eGFR* estimated glomerular filtration rate, *AHD* antihypertensive drugs, *UA* uric acid, *HbA1c* hemoglobin A1c, *FBG* fasting blood glucose, *NLR* neutrophil–lymphocyte ratio, *TC* total cholesterol, *CHD* coronary heart disease, *WBC* white blood cell, *MAP* mean arterial blood pressure, *hs-CRP* high-sensitivity C-reactive protein, *LLD* lipid-lowering drugs, *HGD* hypoglycemic drugs, *PPD* pulse pressure difference, *Cr* creatinine, *Cys* C Cystatin C, *BNP* B-type natriuretic peptide, *ASCVD* atherosclerotic cardiovascular disease

Additional file 1: Table S4 displays the quality evaluation results, which indicate that the overall quality of the cross-sectional studies was acceptable. However, three studies [15, 41, 43] did not adjust for confounding factors, and five studies[31, 34, 37, 39, 41, 43] did not use objective criteria to classify subgroups. The cohort studies scored between 7 and 8 on the NOS scale (Additional file 1: Table S5).

### Association between the TyG index and risk of arterial stiffness

**Fig. 2:**
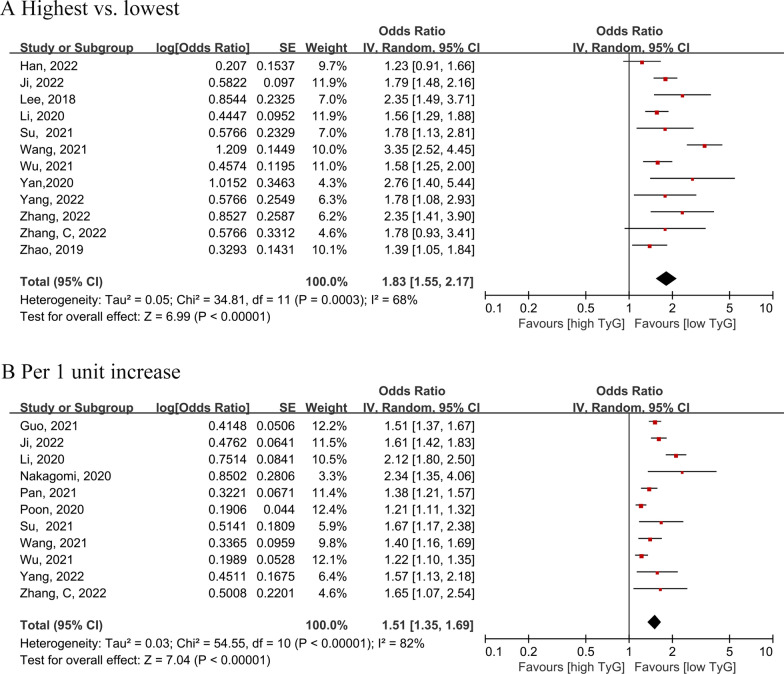
Forest plot of the association between the triglyceride-glucose index (**A** analyzed as categorical variable; **B** analyzed as continuous variable) and the risk of arterial stiffness. The black midline indicates the line of no effect. The diamond indicates the pooled estimate. Red boxes are relative to study size, and the black transverse lines indicate the 95% confidence interval around the effect size estimate.

Twelve studies [9, 14, 29, 36, 39, 40, 44–49] examined the TyG index as a categorical variable to evaluate the risk of arterial stiffness. The highest TyG group were associated with greater risk of arterial stiffness compared to the lowest TyG group (OR = 1.83, 95% CI 1.55–2.17, I^2^ = 68%, τ^2^ = 0.05) (Fig. 2A**)***.* When the TyG index was treated as a continuous variable [14, 28, 36, 40–45, 47, 48], each unit of the TyG index increased the risk of arterial stiffness by 51% (OR = 1.51, 95% CI 1.35–1.69, I^2^ = 82%, τ^2^ = 0.03) (Fig. 2B).

### Association between the TyG index and risk of CAC

**Fig. 3:**
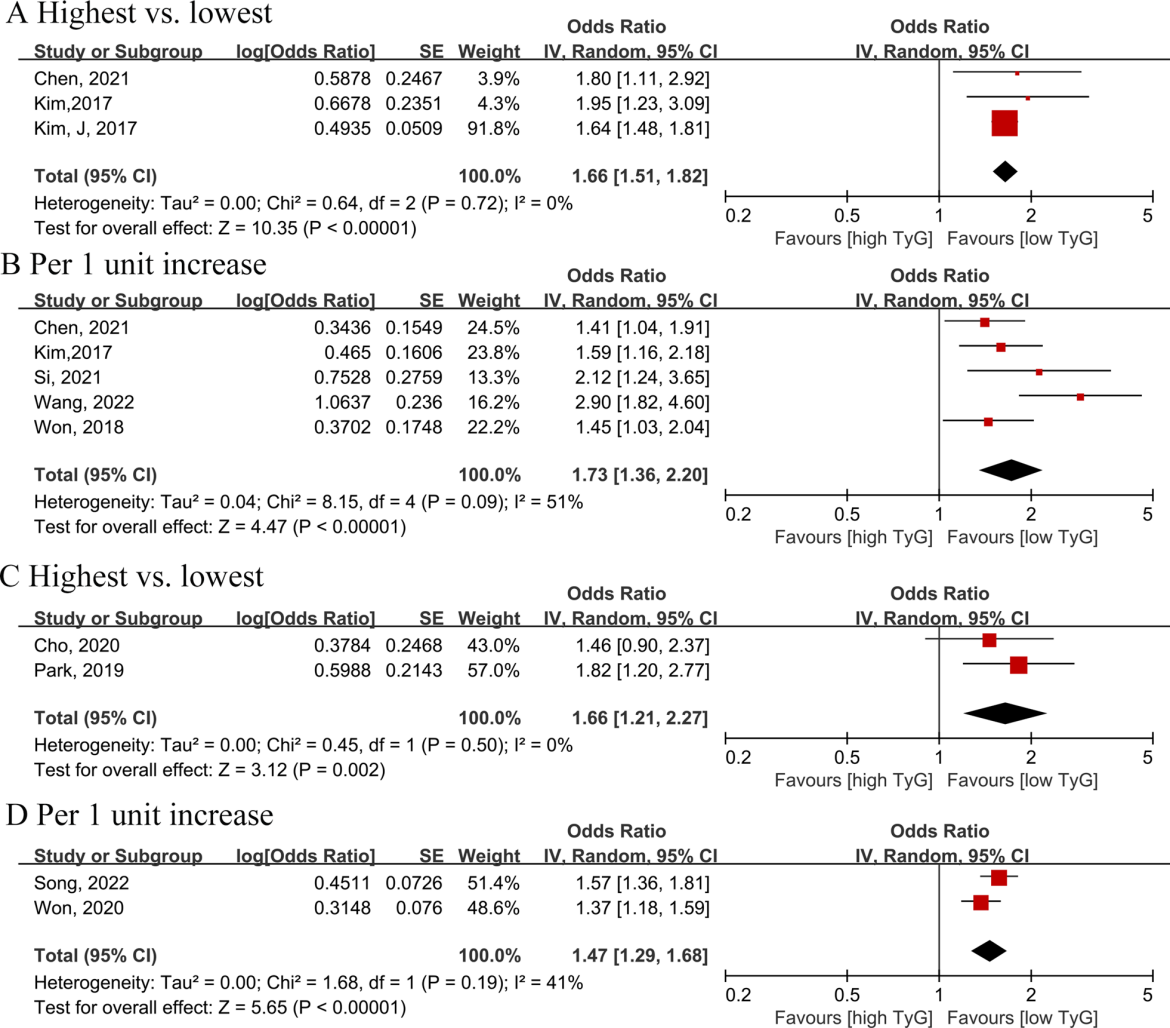
**A** Forest plot of the association between the triglyceride-glucose index (analyzed as a categorical variable) and the risk of coronary artery calcification.** B** Forest plot of the association between the triglyceride-glucose index (analyzed as a continuous variable) and the risk of coronary artery calcification. **C** Forest plot of the association between the triglyceride-glucose index (analyzed as a categorical variable) and the progression of coronary artery calcification. **D** Forest plot of the association between the triglyceride-glucose index (analyzed as a continuous variable) and the progression of coronary artery calcification. The black midline indicates the line of no effect. The diamond indicates the pooled estimate. Red boxes are relative to study size, and the black transverse lines indicate the 95% confidence interval around the effect size estimate.

The pooled results showed a higher TyG index is a risk factor for CAC, increasing the risk by 66% when comparing the highest and lowest categories (OR = 1.66, 95% CI 1.51–1.82, I^2^ = 0, τ^2^ = 0.00) [15, 37, 38] (Fig. 3A). When the TyG index was treated as a continuous variable [15, 31, 33, 34, 38], a positive association was confirmed (OR = 1.73, 95% CI 1.36–2.20, I^2^ = 51%, τ^2^ = 0.04) (Fig. 3B**)**.

Furthermore, we analyzed the relationship between the TyG index and the progression of CAC among four articles [27, 30, 32, 35]. Consistently, we found that the TyG index was associated with the progression of CAC, whether analyzed as category variables (OR = 1.66, 95% CI 1.21–2.27, I^2^ = 0, τ^2^ = 0.00) or continuous variables (OR = 1.47, 95% CI 1.29–1.68, I^2^ = 41%, τ^2^ = 0.00) (Fig. 3C, D).

### Exposure-effect analysis between the TyG index and arterial stiffness

**Fig. 4:**
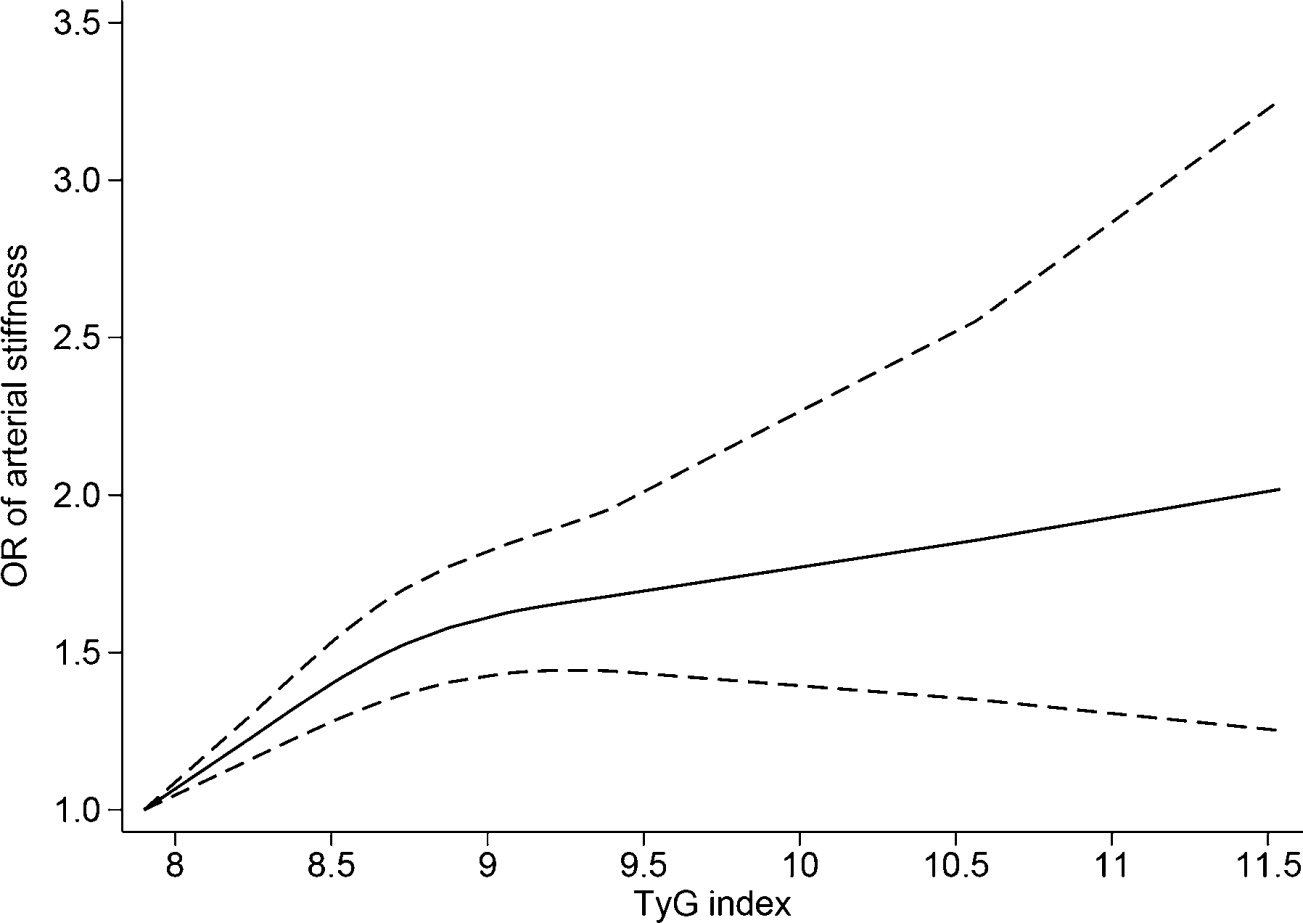
Triglyceride-glucose index and risk of arterial stiffness in nonlinear exposure-effect analysis. The solid line and the dashed lines represent the estimated odd ratio and the 95% confidence interval, respectively

Ten studies [9, 14, 29, 36, 39, 40, 44, 45, 47, 49] were included for the exposure-effect meta-analysis of the TyG index and arterial stiffness. A positive relationship is shown in Fig. 4 with evidence of nonlinearity (P_nonlinearity_ < 0.001). Interestingly, the exposure-effect curve became less steep at TyG index values of approximately 9. The estimated OR derived from the exposure-effect curve is shown in Additional file 1: Table S6.

### Sensitivity analysis and publication bias

We conducted sensitivity analyses for each outcome separately by deleting each study (Additional file 1: Fig. S1). Our results showed that the combined estimated effect of arterial stiffness ranged from 1.64 (95% CI 1.47–1.85) to 1.90 (95% CI 1.59–2.28) when the TyG index was recognized as a categorical variable. When analyzed continuously, the estimated effect ranged from 1.43 (95% CI 1.30–1.57) to 1.55 (95% CI 1.38–1.75), showing that our results are roubst. We also conducted sensitivity analyses of the TyG index with CAC and found stable results.

We assessed publication bias using funnel plots, Egger's test (P = 0.511), and Begg's test (P = 0.631), which indicated relatively low evidence of publication bias (Additional file 1: Fig. S2, S3).

### Subgroup analyses

We performed subgroup analyses for the studies that reported the association between the TyG index (analyzed as a continuous variable) and arterial stiffness, according to mean age, type of study design, sample size, mean body mass index (BMI), and adjustment for confounders (Table 2). The combined OR for individuals with a BMI < 24 was 1.97 (95% CI 1.61–2.42), while the result for those with a BMI ≥ 24 was 1.39 (95% CI 1.26–1.53), suggesting that the TyG index was more closely associated with arterial stiffness in the former group. Moreover, in the subgroups adjusted for confounders, the summary OR changed dramatically in the BMI group, with 1.55 (95% CI 1.38–1.75) for those already adjusted for BMI and 1.21 (95% CI 1.11–1.32) for those not adjusted (P < 0.001). Similar results were also shown in the HDL-C group, suggesting a subgroup difference for the adjustment for BMI or HDL-C.

**Table 2:** Subgroup analysis of TyG and risk of arterial stiffness

Items	Number of studies	ES (95%CI)	P	P^*^_h__(%)_	P^#^
Result of primary analysis	11	1.51 [1.35, 1.69]	$<$ 0.001	82	–
Mean age					
$<$ 60 years	7	1.61 [1.23, 2.11]	$<$ 0.001	93	0.41
$\ge$ 60 years	4	1.42 [1.28, 1.58]	$<$ 0.001	56	–
Study design					
Cohort	3	1.44 [1.22, 1.69]	$<$ 0.001	85	0.46
Cross-sectional	8	1.57 [1.32, 1.87]	$<$ 0.001	83	–
Sample size					
$<$ 4000	6	1.47 [1.25, 1.73]	$<$ 0.001	58	0.73
$\ge$ 4000	5	1.53 [1.30, 1.80]	$<$ 0.001	88	-
Mean BMI					
$<$ 24	3	1.97 [1.61, 2.42]	$<$ 0.001	30	0.01
$\ge$ 24	7	1.39 [1.26, 1.53]	$<$ 0.001	75	–
NR	1	1.65 [1.07, 2.54]	0.02	–	-
Adjustment for confounders					
Age					
Yes	10	1.51 [1.34, 1.70]	$<$ 0.001	83	0.81
No	1	1.57 [1.13, 2.18]	0.007	–	–
Gender					
Yes	8	1.48 [1.29, 1.70]	$<$ 0.001	85	0.50
No	3	1.59 [1.34, 1.88]	$<$ 0.001	18	–
BMI					
Yes	10	1.55 [1.38, 1.75]	$<$ 0.001	77	$<$ 0.001
No	1	1.21 [1.11, 1.32]	$<$ 0.001	–	–
Smoking					
Yes	9	1.55 [1.37, 1.76]	$<$ 0.001	79	0.21
No	2	1.31 [1.04, 1.66]	$<$ 0.001	56	–
HDL-C					
Yes	8	1.66 [1.48, 1.85]	$<$ 0.001	57	$<$ 0.001
No	3	1.25 [1.16, 1.35]	$<$ 0.001	32	–
Medication status					
Yes	5	1.64 [1.43, 1.88]	$<$ 0.001	72	0.02
No	6	1.33 [1.20, 1.48]	$<$ 0.001	55	–
Diabetes					
Yes	4	1.56 [1.17, 2.08]	$<$ 0.001	91	0.72
No	7	1.47 [1.30, 1.66]	$<$ 0.001	73	–
Exercise					
Yes	5	1.64 [1.24, 2.16]	$<$ 0.001	88	0.42
No	6	1.45 [1.29, 1.62]	$<$ 0.001	74	–

*TyG* triglyceride and glucose index, *CI* confidence interval, *BMI* body mass index, *SBP* systolic blood pressure, *HDL-C* high density lipoprotein cholesterol, *NR* not report

*P for within-group heterogeneity, #P for subgroup difference

## Discussion

### Major findings

Our study found that a higher TyG index was associated with an increased risk of arterial stiffness and CAC, regardless of whether the TyG index was analyzed as a categorical variable or continuous variable. Additionally, the TyG index was associated with the progression of CAC. Our exposure-effect meta-analysis also demonstrated a nonlinear positive association between the TyG index and the risk of arterial stiffness.

Previous studies have investigated the relationship between the TyG index and arterial stiffness and CAC. Guo et al. [28] found that the TyG index is independently associated with increased baPWV, a simple and noninvasive method that correlates well with arterial stiffness, in the general Chinese population. Chen et al. [15] reported that a higher TyG index is associated with an increased risk of widespread abdominal aortic CAC. Our findings are consistent with these studies, but our research provides unique insights into the pathogenesis of both arterial stiffness and CAC, suggesting that IR may play a critical role. Furthermore, our study is the first to demonstrate a nonlinear positive relationship between the TyG index and arterial stiffness.

Cardiovascular risk factors or diseases can also impact the TyG level and may potentially confound the association between TyG index and CAC or stiffness. Then we did subgroup analyses stratified by these adjustments. The results showed that our findings remained reliable, regardless of whether we adjusted for age, gender, smoking, obesity, diabetes, exercise status, and medication status.

Elevated plasma glucose has been identified as a potential risk factor for arterial stiffness. For instance, Wang et al. [50] reported a positive association between increased fasting plasma glucose (FPG) levels and the prevalence of arterial stiffness, while Shin et al. [51] showed that even within the normal range, increasing FPG levels were associated with increased risk of arterial stiffness. It is worth nothing that the calculation method of TyG also shows that plasma glucose will affect the measurement of TyG index. Therefore, adjustments for plasma glucose as a confounding factor in the original studies may impact our findings. However, our subgroup analysis revealed that adjustments for diabetes did not significantly affect our results (P-value > 0.05), indicating the credibility of our findings.

Table 2 indicates that the group heterogeneity was small in subgroups adjusted for BMI and HDL-C. For example, I^2^ was 57% for the group adujusted for HDL-C, while I^2^ was 32% for the group that not adjusted, which indicates that the adjustment for BMI and HDL-C may be the source of heterogeneity of the results. Further analysis revealed that obesity is a risk factor for arterial stiffness, and HDL-C is also closely related to the onset of arterial stiffness. Wen et al. [52] found that TG/HDL-C was associated with increased arterial stiffness in both male and female (OR = 1.91, 95% CI: 1.11–3.30, P < 0.05, and 2.91 95% CI: 1.02–8.30, P < 0.05, respectively). This could explain whether adjusted BMI and HDL-C may be the source of outcome heterogeneity.

In our subgroup analysis, we found an interesting association between the TyG index and artificial stiffness in individuals with a BMI < 24 kg/m^2^. This is surprising as obesity is generally considered a risk factor for cardiovascular disease and arterial stiffness. However, caution should be exercised in interpreting this result as the subgroup analysis included only three studies, which may limit the accuracy of the findings. It is important to note that several factors can influence the results. For instance, all three studies in the BMI < 24 kg/m^2^ group were cross-sectional, which may affect the stability of the outcome. Moreover, these studies had adjusted for HDL-C, while some studies in the BMI > 24 kg/m^2^ group did not. This adjustment may have increased the association between the TyG index and the risk of arterial stiffness, as reflected in our findings. Specifically, studies adjusted for HDL-C showed a summary OR of 1.66 (95% CI 1.48–1.85) compared to 1.25 (95% CI 1.16–1.35) in studies without adjustment, indicating an 11% higher risk for arterial stiffness (P < 0.001). Therefore, it is important to conduct more studies to determine how BMI affects the association between the TyG index and arterial stiffness.

It is worth noting that the majority of the included studies were cross-sectional (16 out of 26), and the observational design of the eligible studies does not allow us to establish causality. As such, it remains unclear whether a higher TyG index is linked to the incidence of arterial stiffness and CAC. Further research is needed to address this gap in knowledge.

Several relevant prospective studies have have shed light on the relationship between the TyG index and arterial stiffness and coronary artery CAC. Wu et al. [14] observed a 58% higher incidence of arterial stiffness among participants with an elevated TyG index (HR = 1.58; 95% CI, 1.25–2.01) after adjusting for age, sex, BMI and so on. This longitudinal study involved 6,028 participants who were followed up for 26,839 person-years. Similarly, a study by Won et al. [35] found that the TyG index was strongly associated with the progression of coronary artery CAC in 93,707 asymptomatic Korean adults over a 3.3-year follow-up period. The highest TyG index group had a 37.5% incidence of CAC progression compared to 22.7% in the lowest TyG index group, even after adjusting for age, sex, and BMI. These findings suggest a significant link between the TyG index and the incidence of arterial stiffness and CAC.

### Mechanism

**Fig. 5:**
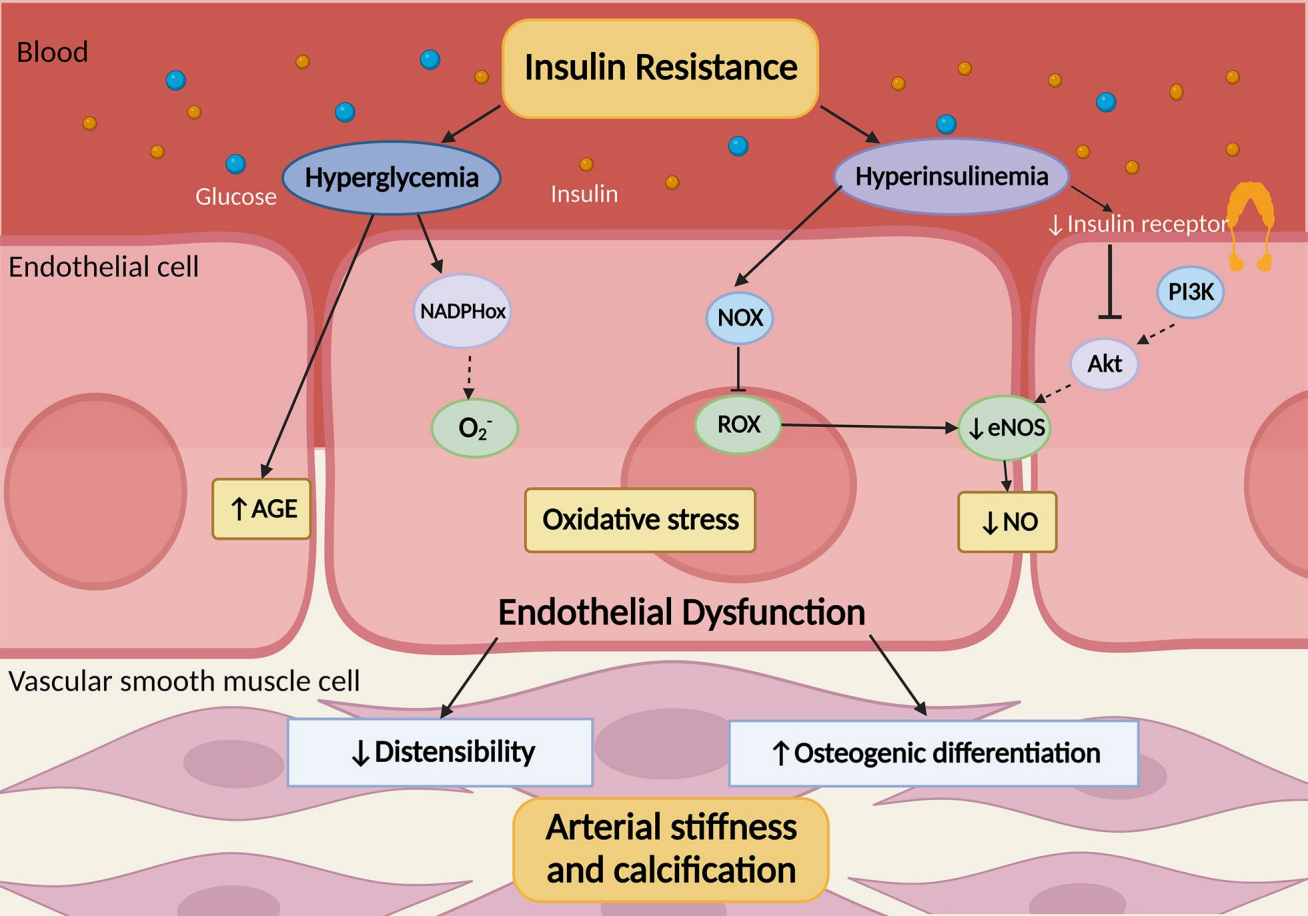
Cartoon describing the potential mechanism of the association between the triglyceride-glucose index and arterial stiffness and coronary artery calcification

Arterial stiffness (arteriosclerosis) and coronary artery calcification (CAC) are two distinct concepts, although they are often associated with each other. According to Mitchell et al. [53], atherosclerosis is a patchy intimal abnormality that is most likely the result of arteriosclerosis, which caused by the increased production of hard load-bearing elements in the arterial wall. Therefore, it is important not to confused these two concepts. However, some of the mechanisms underlying the association between the TyG index and arterial stiffness and CAC are overlapping, and we focus on these common mechanisms (Fig. 5).

One of the main factors that contribute to both arterial stiffness and CAC is IR, which is often accompanied by hyperinsulinemia and hyperglycemia. These metabolic disturbances have been widely recognized as risk factors for arterial stiffness and CAC [54–56]. Hyperinsulinemia can lead to oxidative stress and impaired endothelial cell function, which in turn reduces the bioavailability of nitric oxide [57], causing functional and structural damage to blood vessels, such as reducing the distensibility of the arterial wall (leading to arterial stiffness) [58]. It can also induce osteogenic differentiation and CAC of vascular cells [59]. Moreover, IR can accelerate the accumulation of advanced glycosylation end-products (AGE), which further promote arterial stiffness and CAC [60] Therefore, IR may play a crucial role in the development of arterial stiffness and CAC, which can help explain the association between the TyG index and these cardiovascular outcomes.

### Clinical implication

Currently, the predictive ability of the commonly used clinical index for arterial stiffness and CAC, CACS, remains underwhelming. While CACS measures the calcium content in the coronary artery by CT scan, it does not use all the calcium density information, leading to many patients receiving a zero score [61]. However, studies have reported the TyG index as a surrogate marker of IR and its predictive role in many cardiovascular diseases [62, 63]. Therefore, it may improve the predictive efficiency of arterial stiffness and CAC. Since the TyG index can be obtained simply by regular blood tests, it could serve as an alternative indicator for the occurrence and progression of arterial stiffness and CAC in the future. The diagnostic performance of the TyG index for predicting high baPWV was analyzed by Guo et al. [28], who reported an area under the curve of 0.580 (95% CI 0.565–0.595). The simplicity of its detection suggests that the TyG index might serve as a diagnostic method for arterial stiffness clinically. Moreover, the best cut value for the TyG index was 8.55, which is consistent with the result of the study by Lee et al. [39], showing a positive linear association between the highest quantile of the TyG index (> 8.57) and baPWV. Our exposure-effect analysis found that the positive association between TyG and arterial stiffness increased sharply when the TyG index was approximately 9, and after that, the growth rate of the curve slowed down, implying that approximately 8.6 may be the optimal cut-off point for the TyG index to predict the occurrence of arterial stiffness. Thus, the TyG index may have significant clinical prospects for the diagnosis and prediction of arterial stiffness and CAC, but more research and exploration are needed in the future.

### Limitations

Our results presents the first analysis of the exposure-effect relationship between the TyG index and arterial stiffness and the first exploration of the association between the TyG index and CAC. However, we must acknowledge some limitations. Firstly, the majority of eligible studies were conducted in Asia, which may limit the generalizability of our findings to other populations. Secondly, previous research has demonstrated a direct relationship between the TyG index and other metabolic factors, such as obesity, hypertension, and type 2 diabetes [42, 64]. For instance, studies have confirmed that the TyG index can be independently predict adverse cardiovascular events in patients with diabetes [65], and and our previous research suggests a significant relationship between a high TyG index and the incidence of cardiovascular disease in the general population [8]. Nevertheless, it is unclear whether there is an interaction between diabetes and the association between TyG and arterial stiffness and CAC. Furthermore, although bapwv is a commonly used measure of arterial stiffness in clinical practice, it is a non-specific indicator and cannot be used as an alternative to assessing arterial stiffness. Therefore, further studies are needed to confirm our results.

## Conclusion

Our study showed that the TyG index is associated with an increased risk of arterial stiffness and CAC. Moreover, we observed a positive relationship between the TyG index and arterial stiffness with a nonlinear shape. Nevertheless, we should consider that the results may be influenced by the cross-sectional design and potential confounding factors. Therefore, further investigations are necessary to evaluate the potential of the TyG index as a predictor for arterial stiffness and CAC in addition to existing risk scores.

## Supplementary Information

**Additional file 1**: **Table S1. **PRISMA Checklist. **Table S2**. Search strategy. **Table S3**. Studies excludedwith reasons. **Table S4**. Joanna Briggs Institute critical appraisal checklist applied for included studies. **Table S5**. Quality assessment of the included studies by Newcastle–Ottawa scale. **Table S6**. Odds ratio from the linear dose-response analysis. **Figure S1**. Sensitivity analysis of the association between triglyceride-glucose index and the risk of arterial stiffnessand coronary artery calcification. **Figure S2**. Publication bias detected by funnel plot, Egger’s test and Begg’s test for the association between TyGand the risk of arterial stiffness. **Figure S3**. Publication bias detected by funnel plot, Egger’s test and Begg’s test for the association between TyGand the risk of coronary artery calcification..

## Data Availability

All data generated or analyzed during this study are included in this published article [and its Additional files].

## Abbreviations

CVD: Cardiovascular disease
CAC: Coronary artery calcification
TyG index: Triglyceride and glucose index
IR: Insulin resistance
HOMA-IR: Homeostasis model assessment-insulin resistance
BMI: Body mass index
HR: Hazard ratio
OR: Odds ratio
CI: Confidence interval
NOS: Newcastle‒Ottawa Scale
CACS: Coronary artery calcium score
baPWV: Brachial-ankle pulse wave velocity
