# Supplementary material for: Association between triglyceride glucose index and arterial stiffness and coronary artery calcification: a systematic review and exposure-effect meta-analysis
Source: Cardiovasc Diabetol. 2023 May 13;22:111. doi: 10.1186/s12933-023-01819-2 (PMC10183133; doi:10.1186/s12933-023-01819-2)

**Data Supplement**

**Association between triglyceride glucose index and arterial stiffness and coronary artery calcification: A systematic review and exposure-effect meta-analysis**

Fuwei Liu*, M.D., Qin Ling*, M.D., Shaofeng Xie*, M.D., Yi Xu, M.D., Menglu Liu, Qingwen Hu, M.D., Jianyong Ma, M.D., Zhiwei Yan, MP.T., Yan Gao, MP.T., Yujie Zhao, Wengen Zhu^#6^, Peng Yu^7^, M.D., Jun Luo^1^, M.D., Xiao Liu^#8^

Author Affiliations：

Department of Cardiology, the Affiliated Ganzhou Hospital of Nanchang University, Jiangxi, China (F.W.L, S.F.X., J.L); The Second Clinical Medical College of Nanchang University, The Second Affiliated Hospital of Nanchang University, Nanchang, China (Q.L., S.F.X., Y.X., Q.W.H.); Department of Cardiology, Seventh People’s Hospital of Zhengzhou, Zhengzhou, Henan, China (M.L.L., Y.J.Z.); Department of Pharmacology and Systems Physiology, University of Cincinnati College of Medicine, Cincinnati, USA (J.Y.M.); Department of Sports Rehabilitation, College of Human Kinesiology, Shenyang Sport University, Shenyang, China (Z.W.Y., Y.G.); Department of Cardiology, the First Affiliated Hospital of Sun Yat-Sen University, Guangzhou, Guangdong, China (W.G.Z.); Department of Endocrine, the Second Affiliated Hospital of Nanchang University, Nanchang, Jiangxi, China (P.Y.); Department of Cardiology, the Sun Yat-Sen Memorial Hospital of Sun Yat-Sen University, Guangzhou, Guangdong, China (X.L.).

* These authors contributed equally to this work as co-first authors.

^#^ These authors are senior authors.

Correspondence to Prof Jun Luo, Department of Cardiology, the Affiliated Ganzhou Hospital of Nanchang University, Jiangxi, China; Email: luojun202212@163.com

**Table of Contents**

[Table S1. PRISMA Checklist. 5](#_Toc29793)

[Table S2. Search strategy 8](#_Toc25437)

[Table S3. Studies excluded (n=6) with reasons 10](#_Toc21761)

[Table S4. Joanna Briggs Institute critical appraisal checklist applied for included studies. 11](#_Toc26683)

[Table S5. Quality assessment of the included studies by Newcastle–Ottawa scale. 12](#_Toc19015)

[Table S6. Odds ratio from the linear dose-response analysis 13](#_Toc5221)

[Figure S1. Sensitivity analysis of the association between triglyceride-glucose index and the risk of arterial stiffness. 14](#_Toc23286)

[Figure S2. Publication bias detected by funnel plot, Egger’s test and Begg’s test for the association between TyG 15](#_Toc30578)

[Figure S3. Publication bias detected by funnel plot, Egger’s test and Begg’s test for the association between TyG (analyzed as a continuous variable) and the risk of coronary artery calcification 16](#_Toc29859)

# Table S1. PRISMA Checklist.

| **Section/topic** | **#** | **Checklist item** | **Reported on page #** |
| --- | --- | --- | --- |
| **TITLE** | | |  |
| Title | 1 | Identify the report as a systematic review, meta-analysis, or both. | 1 |
| **ABSTRACT** | | |  |
| Structured summary | 2 | Provide a structured summary including, as applicable: background; objectives; data sources; study eligibility criteria, participants, and interventions; study appraisal and synthesis methods; results; limitations; conclusions and implications of key findings; systematic review registration number. | 3 |
| **INTRODUCTION** | | |  |
| Rationale | 3 | Describe the rationale for the review in the context of what is already known. | 5 |
| Objectives | 4 | Provide an explicit statement of questions being addressed with reference to participants, interventions, comparisons, outcomes, and study design (PICOS). | 5 |
| **METHODS** | | |  |
| Protocol and registration | 5 | Indicate if a review protocol exists, if and where it can be accessed (e.g., Web address), and, if available, provide registration information including registration number. | 6 |
| Eligibility criteria | 6 | Specify study characteristics (e.g., PICOS, length of follow-up) and report characteristics (e.g., years considered, language, publication status) used as criteria for eligibility, giving rationale. | 7 |
| Information sources | 7 | Describe all information sources (e.g., databases with dates of coverage, contact with study authors to identify additional studies) in the search and date last searched. | 6 |
| Search | 8 | Present full electronic search strategy for at least one database, including any limits used, such that it could be repeated. | 6 |
| Study selection | 9 | State the process for selecting studies (i.e., screening, eligibility, included in systematic review, and, if applicable, included in the meta-analysis). | 7 |
| Data collection process | 10 | Describe method of data extraction from reports (e.g., piloted forms, independently, in duplicate) and any processes for obtaining and confirming data from investigators. | 8 |
| Data items | 11 | List and define all variables for which data were sought (e.g., PICOS, funding sources) and any assumptions and simplifications made. | 7 |
| Risk of bias in individual studies | 12 | Describe methods used for assessing risk of bias of individual studies (including specification of whether this was done at the study or outcome level), and how this information is to be used in any data synthesis. | 9 |
| Summary measures | 13 | State the principal summary measures (e.g., risk ratio, difference in means). | 9 |
| Synthesis of results | 14 | Describe the methods of handling data and combining results of studies, if done, including measures of consistency (e.g., I^2^) for each meta-analysis. | 9 |
| Risk of bias across studies | 15 | Specify any assessment of risk of bias that may affect the cumulative evidence (e.g., publication bias, selective reporting within studies). | 9 |
| Additional analyses | 16 | Describe methods of additional analyses (e.g., sensitivity or subgroup analyses, meta-regression), if done, indicating which were pre-specified. | 9 |
| **RESULTS** | | |  |
| Study selection | 17 | Give numbers of studies screened, assessed for eligibility, and included in the review, with reasons for exclusions at each stage, ideally with a flow diagram. | 9 |
| Study characteristics | 18 | For each study, present characteristics for which data were extracted (e.g., study size, PICOS, follow-up period) and provide the citations. | 10 |
| Risk of bias within studies | 19 | Present data on risk of bias of each study and, if available, any outcome level assessment (see item 12). | 12 |
| Results of individual studies | 20 | For all outcomes considered (benefits or harms), present, for each study: (a) simple summary data for each intervention group (b) effect estimates and confidence intervals, ideally with a forest plot. | 11 |
| Synthesis of results | 21 | Present results of each meta-analysis done, including confidence intervals and measures of consistency. | 11 |
| Risk of bias across studies | 22 | Present results of any assessment of risk of bias across studies (see Item 15). | 12 |
| Additional analysis | 23 | Give results of additional analyses, if done (e.g., sensitivity or subgroup analyses, meta-regression [see Item 16]). | 12 |
| **DISCUSSION** | | |  |
| Summary of evidence | 24 | Summarize the main findings including the strength of evidence for each main outcome; consider their relevance to key groups (e.g., healthcare providers, users, and policy makers). | 13 |
| Limitations | 25 | Discuss limitations at study and outcome level (e.g., risk of bias), and at review-level (e.g., incomplete retrieval of identified research, reporting bias). | 18 |
| Conclusions | 26 | Provide a general interpretation of the results in the context of other evidence, and implications for future research. | 19 |
| **FUNDING** | | |  |
| Funding | 27 | Describe sources of funding for the systematic review and other support (e.g., supply of data); role of funders for the systematic review. | 19 |

*From:*  Moher D, Liberati A, Tetzlaff J, Altman DG, The PRISMA Group (2009). Preferred Reporting Items for Systematic Reviews and Meta-Analyses: The PRISMA Statement. PLoS Med 6(7): e1000097. doi:10.1371/journal.pmed1000097

For more information, visit: **www.prisma-statement.org**.

# Table S2. Search strategy

| Datebase | Search | Query |
| --- | --- | --- |
| PubMed database | #1 | ((Coronary artery calcium score) OR (CACS) OR (Calcification)) OR ((Stiffness) OR (baPWV) OR (brachial-ankle pulse wave velocity) OR (Pulse wave velocity) OR (Carotid-femoral pulse wave velocity) OR (cfPWV) OR (Cardiac-Ankle Vascular Index) OR (CAVI)) |
|  | #2 | ((((TyG index) OR (triglyceride-glucose index)) OR (triglyceride and glucose index)) OR (triglyceride glucose index)) OR (triacylglycerol glucose index) |
|  | #3 | #1 AND #2 |
| Embase database | #1 | 'arterial stiffness'/exp |
|  | #2 | 'calcification'/exp |
|  | #3 | 'coronary artery calcium score'/exp |
|  | #4 | 'brachial-ankle pulse wave velocity'/exp |
|  | #5 | 'Pulse wave velocity'/exp |
|  | #6 | 'Carotid-femoral pulse wave velocity'/exp |
|  | #7 | 'Cardiac-Ankle Vascular Index'/exp |
|  | #8 | #1 OR #2 OR #3 OR #4 OR #5 OR #6 OR #7 |
|  | #9 | 'tyg index' OR 'triglyceride-glucose index' OR 'triglyceride and glucose index' OR 'triglyceride glucose index' OR 'triacylglycerol glucose index' |
|  | #10 | #8 AND #9 |
| Cochrane library | #1 | MeSH descriptor: [Vascular Stiffness] explode all trees |
|  | #2 | (Vascular Stiffness):ti,ab,kw |
|  | #3 | MeSH descriptor: [Vascular Calcification] explode all trees |
|  | #4 | (Vascular Calcification):ti,ab,kw |
|  | #5 | (coronary artery calcium score):ti,ab,kw |
|  | #6 | (brachial-ankle pulse wave velocity):ti,ab,kw |
|  | #7 | MeSH descriptor: [Pulse wave velocity] explode all trees |
|  | #8 | (Pulse wave velocity):ti,ab,kw |
|  | #9 | (Carotid-femoral pulse wave velocity):ti,ab,kw |
|  | #10 | (Cardiac-Ankle Vascular Index):ti,ab,kw |
|  | #11 | (triglyceride and glucose index): ti,ab,kw |
|  | #12 | #1 OR #2 OR #3 OR #4 OR # 5 OR #6 OR #7 OR #8 OR #9 |
|  | #13 | #10 AND #11 |

# Table S3. Studies excluded (n=6) with reasons

| **Studies excluded** | **Reasons (according to PICOS)** |
| --- | --- |
| Wu, 2021[1] | Without target data set: Not providing OR, HR or RR |
| Baydar, 2021[2] | Without target data set: Not providing OR, HR or RR |
| Chiu, 2021[3] | Without target data set: Not providing OR, HR or RR |
| Mehta, 2021[4] | Without target data set: Not providing OR, HR or RR |
| Won, 2018[5] | Without target data set: Not providing OR, HR or RR |
| Lambrinoudaki, 2018[6] | Without target data set: Not providing OR, HR or RR |

[1] Z. Wu, D. Zhou, Y. Liu, Z. Li, J. Wang, Z. Han, X. Miao, X. Liu, X. Li, W. Wang, X. Guo, and L. Tao, Association of TyG index and TG/HDL-C ratio with arterial stiffness progression in a non-normotensive population. Cardiovascular diabetology 20 (2021) 134.

[2] O. Baydar, A. Kilic, J. Okcuoglu, Z. Apaydin, and M.M. Can, The Triglyceride-Glucose Index, a Predictor of Insulin Resistance, Is Associated With Subclinical Atherosclerosis. Angiology 72 (2021) 994-1000.

[3] T.-H. Chiu, H.-J. Tsai, H.-Y.C. Chiou, P.-Y. Wu, J.-C. Huang, and S.-C. Chen, A high triglyceride-glucose index is associated with left ventricular dysfunction and atherosclerosis. Int J Med Sci 18 (2021) 1051-1057.

[4] R. Mehta, N.E. Antonio-Villa, O.Y. Bello-Chavolla, A.J. Martagón, D. Elias-López, A. Vargas-Vázquez, D.V. Gómez-Velasco, P. Almeda-Valdés, L. Muñoz-Hernandez, I. Cruz-Bautista, and C.A. Aguilar-Salinas, Association between insulin resistance and arterial stiffness in Mexican patients without type 2 diabetes. Gaceta medica de Mexico 157 (2021) 522-530.

[5] K.B. Won, G.M. Park, S.E. Lee, I.J. Cho, H.C. Kim, B.K. Lee, and H.J. Chang, Relationship of insulin resistance estimated by triglyceride glucose index to arterial stiffness. Lipids in health and disease 17 (2018) 268.

[6] I. Lambrinoudaki, M.V. Kazani, E. Armeni, G. Georgiopoulos, K. Tampakis, D. Rizos, A. Augoulea, G. Kaparos, A. Alexandrou, and K. Stamatelopoulos, The TyG Index as a Marker of Subclinical Atherosclerosis and Arterial Stiffness in Lean and Overweight Postmenopausal Women. Heart, lung & circulation 27 (2018) 716-724.

# Table S4. Joanna Briggs Institute critical appraisal checklist applied for included studies.

| Study | Sample was representative? | Participants appropriately recruited? | Sample size was adequate? | Study subjects and the setting described | Data analysis conducted | Objective, standard criteria, reliably used? | Appropriate statistical analysis used? | Confounding factors/ subgroups/ differences identified and accounted? | Subpopulations identified using objective criteria |
| --- | --- | --- | --- | --- | --- | --- | --- | --- | --- |
| Kim, 2017 | Yes | Yes | Yes | Yes | Yes | Yes | Yes | Yes | Yes |
| Kim, J, 2017 | Yes | Yes | Yes | Yes | Yes | Yes | Yes | Yes | Unclear |
| Lee, 2018 | Yes | Yes | Yes | Yes | Yes | Yes | Yes | Yes | Unclear |
| Zhao, 2019 | Yes | Yes | Yes | Yes | Yes | Yes | Yes | Unclear | Yes |
| Li, 2020 | Yes | Yes | Yes | Yes | Yes | Yes | Yes | Yes | Yes |
| Poon, 2020 | Yes | Yes | Yes | Yes | Yes | Yes | Yes | No | Unclear |
| Nakagomi, 2020 | Yes | Yes | Yes | Yes | Yes | Yes | Yes | No | Unclear |
| Pan, 2021, | Yes | Yes | Yes | Yes | Yes | Yes | Yes | Yes | Yes |
| Chen, 2021 | Yes | Yes | Yes | Yes | Yes | Yes | Yes | No | Yes |
| Su,2021 | Yes | Yes | Yes | Yes | Yes | Yes | Yes | Yes | Yes |
| Wang, 2021 | Yes | Yes | Yes | Yes | Yes | Yes | Yes | Yes | Yes |
| Zhang, C, 2022 | Yes | Yes | Yes | Yes | Yes | Yes | Yes | Yes | Yes |
| Yang, 2022 | Yes | Yes | Yes | Yes | Yes | Yes | Yes | Yes | Yes |
| Zhang, 2022 | Yes | Yes | Yes | Yes | Yes | Yes | Yes | Yes | Yes |
| Ji, 2022 | Yes | Yes | Yes | Yes | Yes | Yes | Yes | Unclear | Yes |
| Yan, 2022 | Yes | Yes | Yes | Yes | Yes | Yes | Yes | Yes | Unclear |

# Table S5. Quality assessment of the included studies by Newcastle–Ottawa scale.

| Study | Selection | | | | Comparability | Outcome | | | Total |
| --- | --- | --- | --- | --- | --- | --- | --- | --- | --- |
|  | Exposed cohort | Nonexposed cohort | Ascertainment of exposure | Outcome of interest |  | Assessment of outcome | Length of follow-up | Adequacy of follow-up |  |
| Won, 2018 | * | * | * | * |  | * | * | * | 7 |
| Park,2019 | * | * | * | * |  | * | * | * | 7 |
| Cho, 2020 | * | * | * | * | * | * | * | * | 8 |
| Won, 2020 | * | * | * | * | * | * | * | * | 8 |
| Guo,2021 | * | * | * | * | * | * | * |  | 7 |
| Wu, 2021 | * | * | * | * |  | * | * | * | 7 |
| Si, 2021 | * | * | * | * | * | * | * | * | 8 |
| Wang,2022 | * | * | * | * | * | * | * | * | 8 |
| Song, 2022 | * | * | * | * | * | * | * | * | 8 |
| Han, 2022 | * | * | * | * | * | * | * | * | 8 |

Comparability: Age = *, Other control factors = *; Adequacy of follow-up: Follow-up rate≥80% = *.

# Table S6. Odds ratio from the linear dose-response analysis

| **Outcome**  **TyG index** | **Risk of arterial stiffness** |
| --- | --- |
|  | **OR (95%CI)** |
| 7.90 | (1.00-1.00) |
| 8.25 | (1.16-1.31) |
| 8.33 | (1.20-1.38) |
| 8.39 | (1.23-1.44) |
| 8.510 | (1.28-1.54) |
| 8.64 | (1.34-1.64) |
| 8.72 | (1.36-1.69) |
| 8.88 | (1.41-1.77) |
| 9.06 | (1.43-1.84) |
| 9.12 | (1.44-1.86) |
| 9.26 | (1.44-1.91) |
| 9.39 | (1.44-1.95) |
| 10.56 | (1.35-2.55) |
| 11.54 | (1.25-3.26) |
| Pnon-linearity | 0.0002 |

# Figure S1. Sensitivity analysis of the association between triglyceride-glucose index and the risk of arterial stiffness (A:category analysis; B: continuity analysis) and coronary artery calcification (C:category analysis; D: continuity analysis).

**
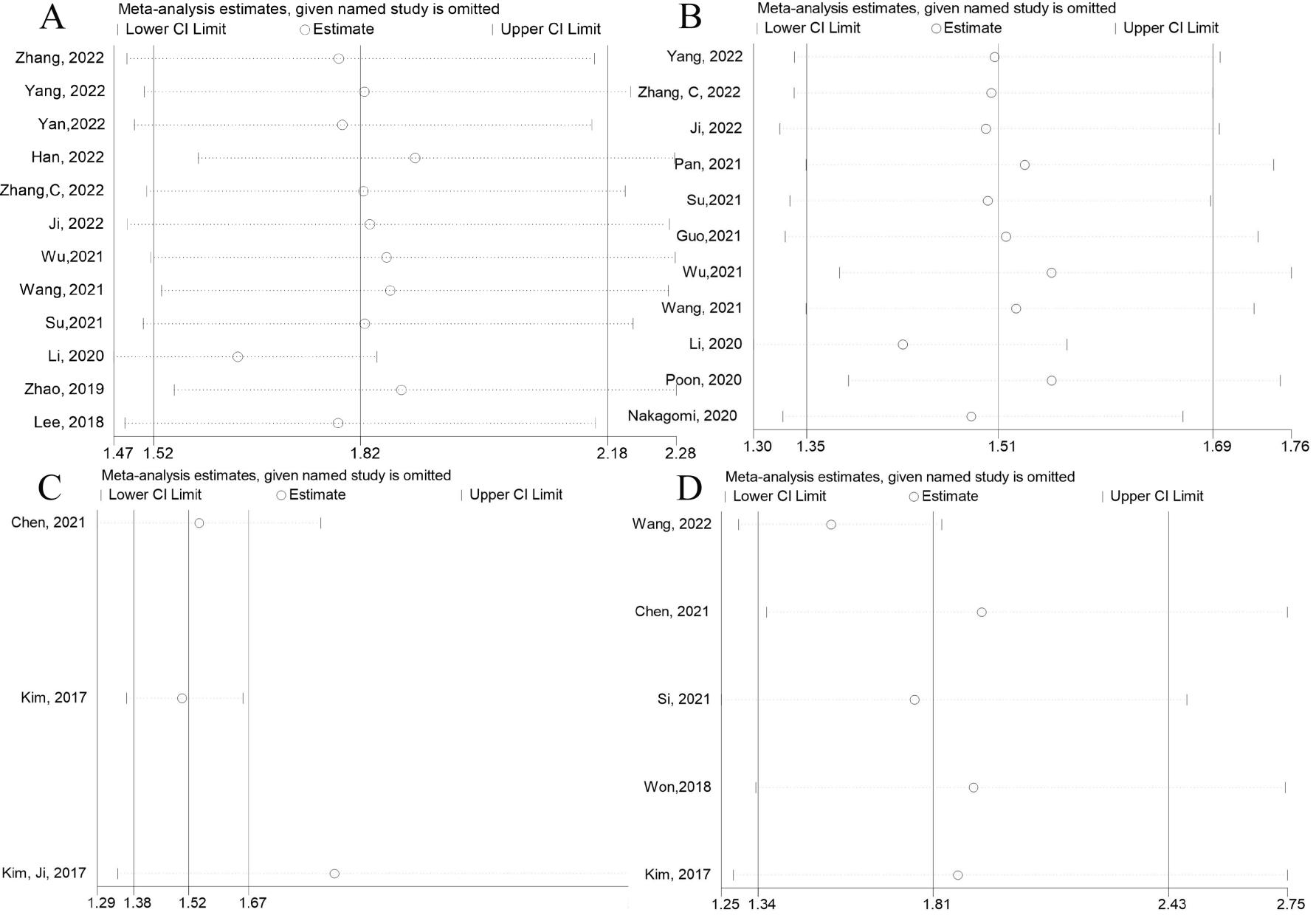
**

# Figure S2. Publication bias detected by funnel plot, Egger’s test and Begg’s test for the association between TyG (analyzed as a categorical variable) and the risk of arterial stiffness(A: Funnel plot; B: Egger’s test; C: begg’s test).


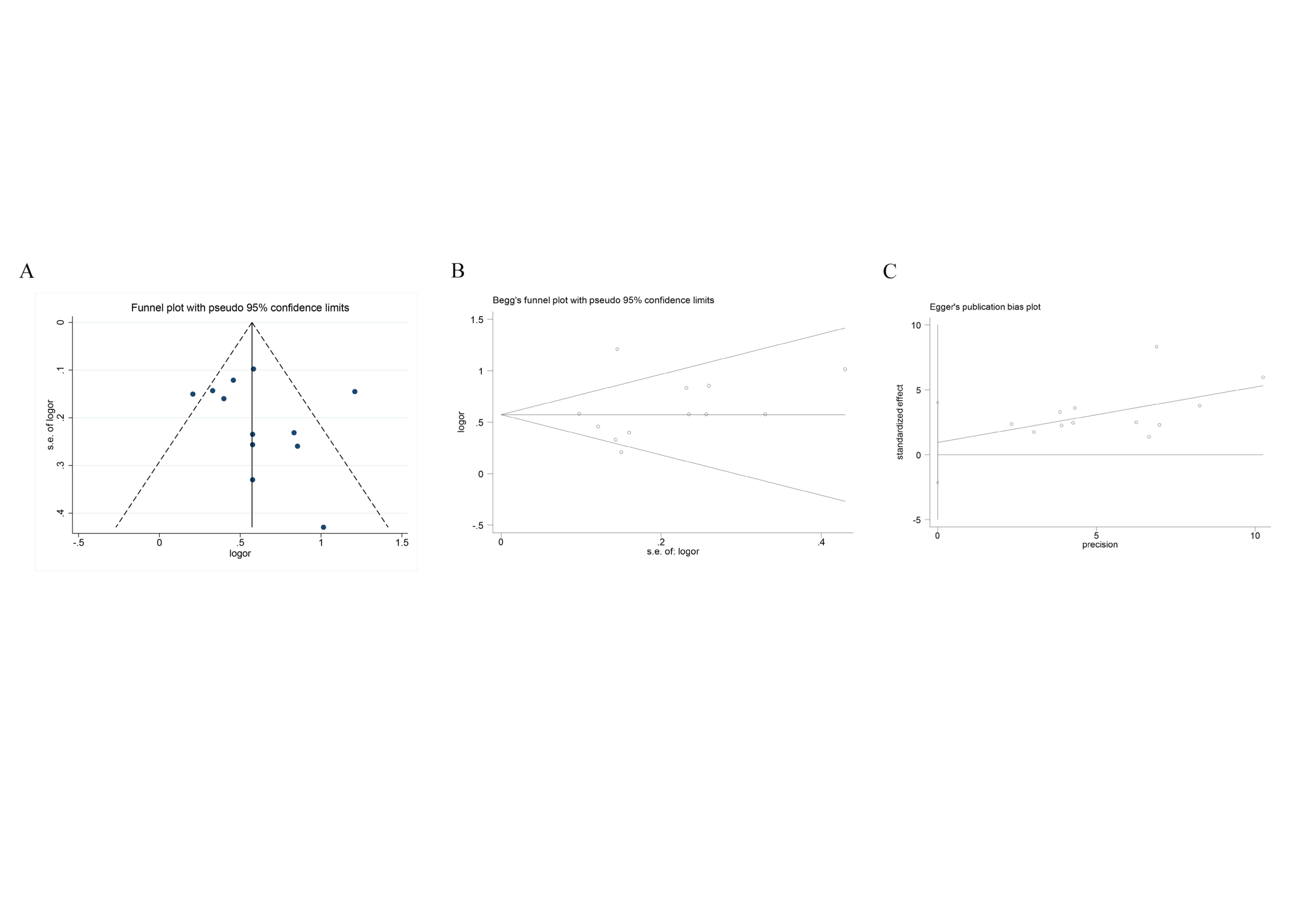


# Figure S3. Publication bias detected by funnel plot, Egger’s test and Begg’s test for the association between TyG (analyzed as a continuous variable) and the risk of coronary artery calcification.(A: Funnel plot; B: Egger’s test; C: begg’s test)


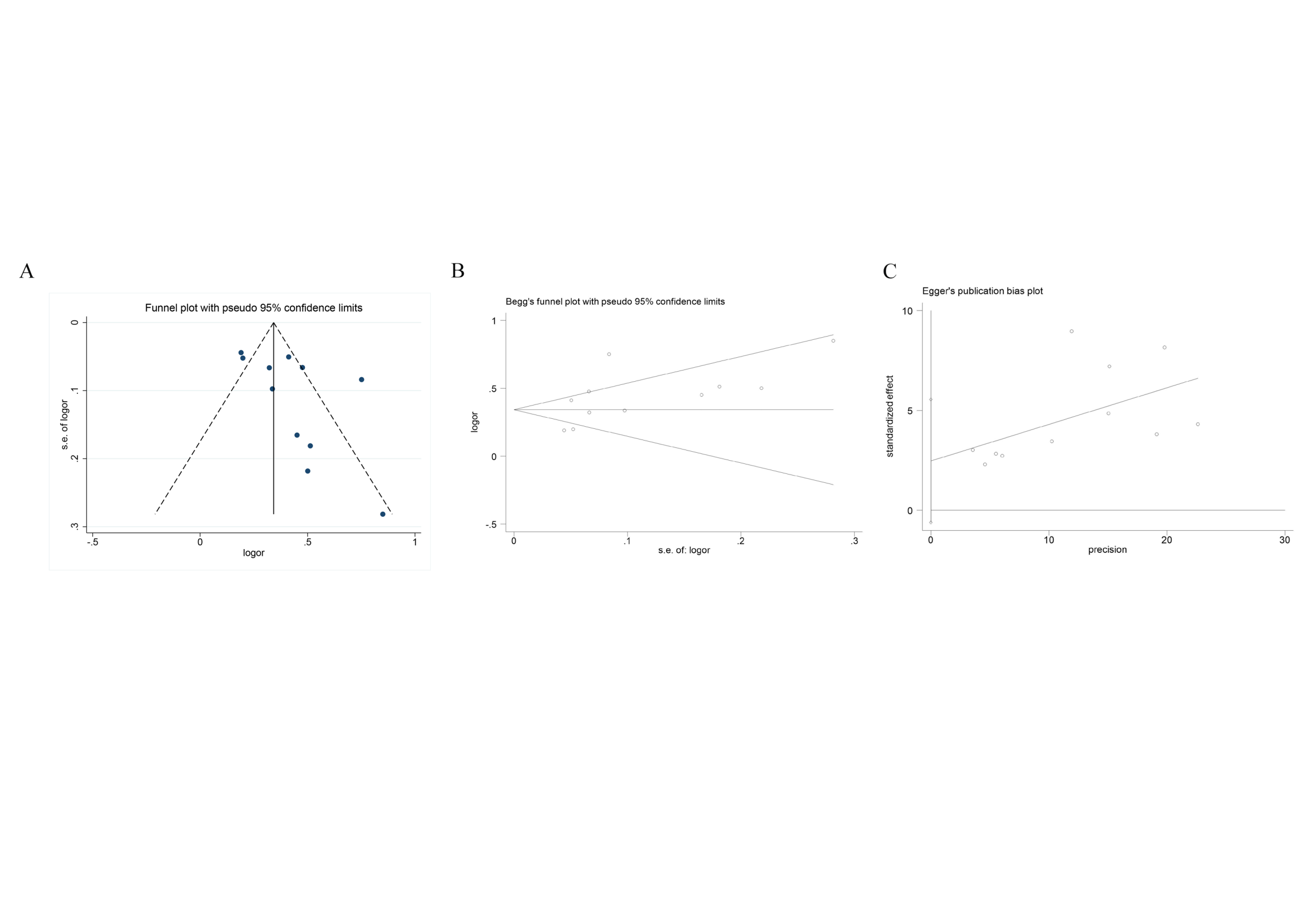

Supplement: Supplementary file 1 — Additional file 1: Table S1. PRISMA Checklist. Table S2. Search strategy. Table S3. Studies excludedwith reasons. Table S4. Joanna Briggs Institute critical appraisal checklist applied for included studies. Table S5. Quality assessment of the included studies by Newcastle–Ottawa scale. Table S6. Odds ratio from the linear dose-response analysis. Figure S1. Sensitivity analysis of the association between triglyceride-glucose index and the risk of arterial stiffnessand coronary artery calcification. Figure S2. Publication bias detected by funnel plot, Egger’s test and Begg’s test for the association between TyGand the risk of arterial stiffness. Figure S3. Publication bias detected by funnel plot, Egger’s test and Begg’s test for the association between TyGand the risk of coronary artery calcification.. [file 12933_2023_1819_MOESM1_ESM.docx]
